# Ultra-wide-field retinal imaging in the management of non-infectious retinal vasculitis

**DOI:** 10.1186/1869-5760-3-30

**Published:** 2013-02-11

**Authors:** Henry A Leder, John P Campbell, Yasir J Sepah, Theresa Gan, James P Dunn, Elham Hatef, Brian Cho, Mohamed Ibrahim, Millena Bittencourt, Roomasa Channa, Diana V Do, Quan Dong Nguyen

**Affiliations:** 1 Retinal Imaging Research and Reading Center, Wilmer Eye Institute, Johns Hopkins Hospital, Johns Hopkins University, Maumenee 745, 600 North Wolfe Street, Baltimore, MD, 21287, USA; 2 Department of Ophthalmology, Albert Einstein College of Medicine, Yeshiva University, Montefiore Medical Center, 3332 Rochambeau Avenue, Centennial Building, Bronx, NY, 10467, USA; 3 Casey Eye Institute, Oregon Health and Sciences University, 3375 SW Terwilliger Blvd., Portland, OR, 97239, USA; 4 Division of Ocular Immunology, Wilmer Eye Institute, Johns Hopkins Hospital, Johns Hopkins University, Maumenee 119, 600 North Wolfe Street, Baltimore, MD, 21287, USA; 5 Stanley M. Truhlsen Eye Institute, University of Nebraska Medical Center, Omaha, NE, 68198, USA

**Keywords:** Non-infectious retinal vasculitis, Fluorescein angiography, Ultra-wide-field imaging

## Abstract

**Background:**

The purpose of this study is to describe and quantify the benefit of ultra-wide-field imaging and fluorescein angiography (FA) in the management of non-infectious retinal vasculitis. In this prospective observational cohort series, patients with non-infectious retinal vasculitis were evaluated and enrolled by four investigators from the Divisions of Retina and Ocular Immunology at the Wilmer Eye Institute. In each patient, disease activity and the need for management changes were assessed, based on clinical examination with or without standard (60°) imaging and then with the addition of ultra-wide-field pseudo-color scanning laser ophthalmoscope (SLO) images and FA using the Optos ultra-wide-field SLO (Optos Panoramic 200MA™, Optos PLC, Dunfermline, Scotland, UK). A standardized questionnaire was completed by each investigator at the time of the clinical evaluation.

The *primary* outcome was the percentage of patients whose management was changed by clinical examination and standard FA, compared with clinical examination plus ultra-wide-field imaging. The *secondary* outcome was the percentage of patients whose disease was determined to be active based on each modality.

**Results:**

Seventy-one visits from 23 patients were reviewed and analyzed. Based on examination plus ultra-wide-field imaging and ultra-wide-field angiography, disease activity was detected in 48/71 (68%) compared with 32/71 (45%) based on examination and standard FA (*P* = 0.0095). Based on the clinical examination alone, the decision to alter management was made in 4 of 71 visits (6%), and an additional 3 of 71 (4%) based on simulated standard FA. The addition of ultra-wide-field SLO pseudo-color images altered management in an additional 10/71 visits (14%), and 36/71 (51%) with the addition of ultra-wide-field FA.

**Conclusions:**

Ultra-wide-field fluorescein imaging and angiography can provide additional information that may be important and relevant in the management of retinal vasculitis.

## Background

Non-infectious retinal vasculitis is a challenging disease to treat. Treatment must be titrated to minimize side effects while preserving vision. Disease activity is monitored through a combination of patient symptoms, clinical examination (including slit lamp, indirect ophthalmoscopy, and direct contact lens biomicroscopy), and retinal imaging studies [1]. Vascular leakage, an important element of retinal vasculitis, is best appreciated and detected with fluorescein angiography (FA). Conventional retinal photography, including FA, is limited in its field of view. Most fundus cameras can capture only 30° to 60° of the fundus at a time. While sweeps of the retina can be performed to sample larger areas of the retina, such an approach cannot image the entire retina simultaneously. The limited view makes it difficult to correctly locate peripheral lesions and to confirm/compare changes over time. The clinician is left relying on diagrams in clinic charts, standard photographs, and memory to assess disease progression.

There are several models of wide-field imaging devices. The Optos P200 (Optos PLC, Dunfermline, Scotland, UK) uses a scanning laser ophthalmoscope (SLO) to image 200° of the retina at a time. It is also capable of fluorescein angiography transiting the same 200° area. Such capabilities make it ideal for monitoring progressive peripheral retinal vascular diseases and may be helpful in the clinical management of posterior uveitis [2,3].

Several case reports have found that ultra-wide-field imaging with FA is well tolerated by patients, can image through small pupils, and can significantly contribute to the management of retinal disease, though there can be a number of imaging artifacts that interfere with image interpretation [2]. It can be useful in a wide variety of retinal vascular disorders including uveitis, cytomegalovirus retinitis, vein occlusions, diabetes, as well as non-vascular conditions such as retinal detachment, retinal tumors, and choroidal tumors. However, these publications are limited to retrospective case reports or series [4-15].

Kaines et al. reviewed five cases of posterior uveitis seen at their institution and identified several attributes of the SLO ultra-wide-field imaging system that aided in the diagnosis and management of their patients [3]. They noted that the high-resolution images allowed clear documentation of peripheral retinal lesions and greatly eased longitudinal comparisons for disease activity and progression. Areas of neovascularization and non-perfusion were easily identified, aiding targeted pan-retinal photocoagulation. In several patients, they also noted peripheral angiographic findings that suggested disease activity in the absence of clinical evidence of the disease.

The index study employed the use of ultra-wide-field imaging modality prospectively in the management of patients with non-infectious retinal vasculitis to determine whether the added information provided by the ultra-wide-field images would alter management compared with standard examination and standard (60°) imaging.

## Results

Seventy-one visits of 23 consented patients were analyzed in this prospective observational case series. All patients had ultra-wide-field imaging following their clinical examination. Associated diagnoses of the 23 patients prior to enrollment into the study included the following: 6 with panuveitis, 3 with biopsy-proven sarcoidosis, 3 with intermediate uveitis, 2 with Adamantiades-Behcet’s disease, 1 with Wegener’s disease, and 8 with no associated syndrome (idiopathic).

**Figure 1:**
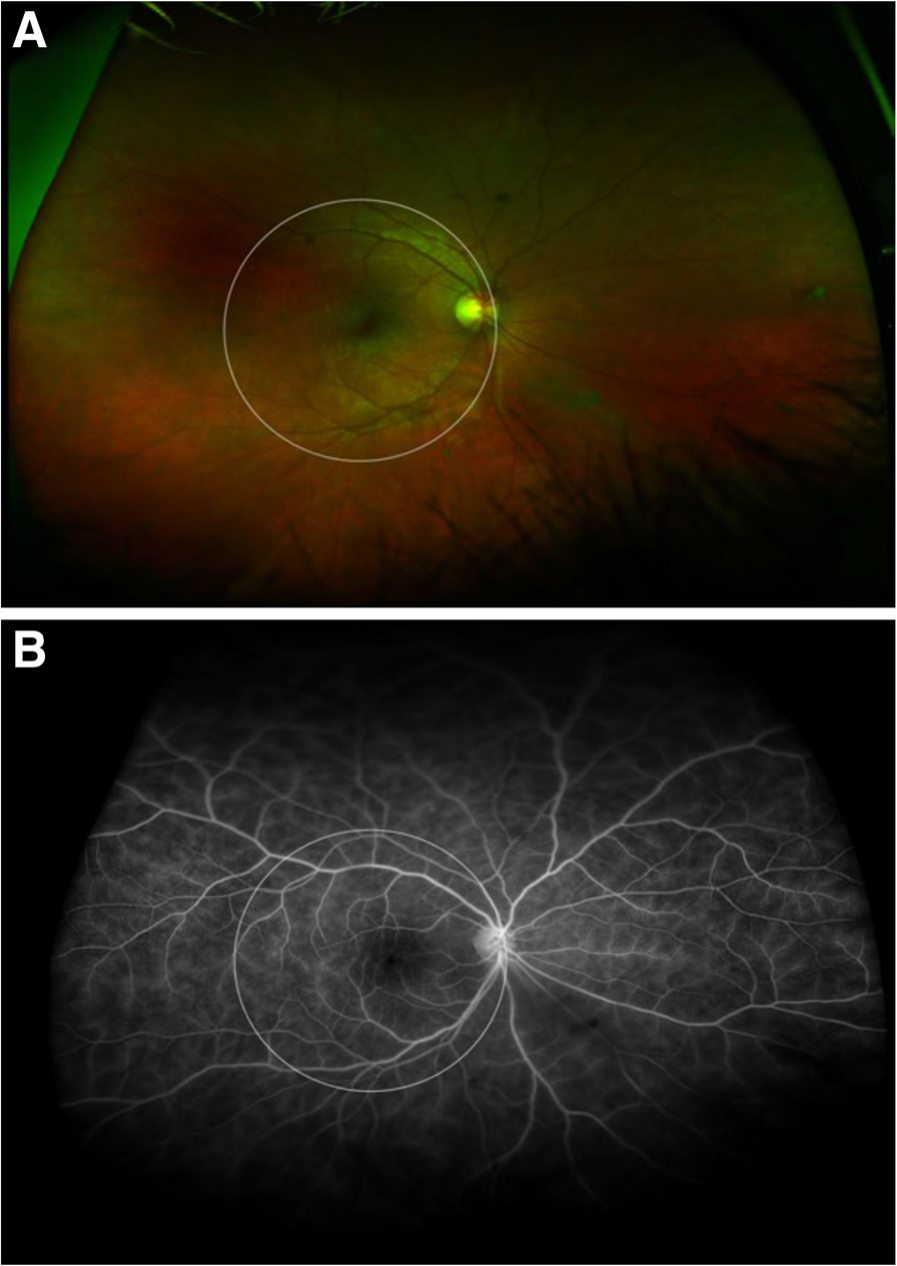
**Color photograph and fluorescein angiography.** (**A**) Wide-field color image and (**B**) corresponding fluorescein angiography of a 14-year-old boy with intermediate uveitis and retinal vasculitis. The white circle simulates the area covered by a typical 60° view.

Figure 1 demonstrates how the simulated FA and clinical examination may detect disease activity (mild perifoveal and perivascular leakage); however, additional findings seen on ultra-wide-field imaging (significant diffuse, peripheral vascular leakage) may affect the investigators’ overall assessment of disease activity and hence may alter plans of management.

**Figure 2:**
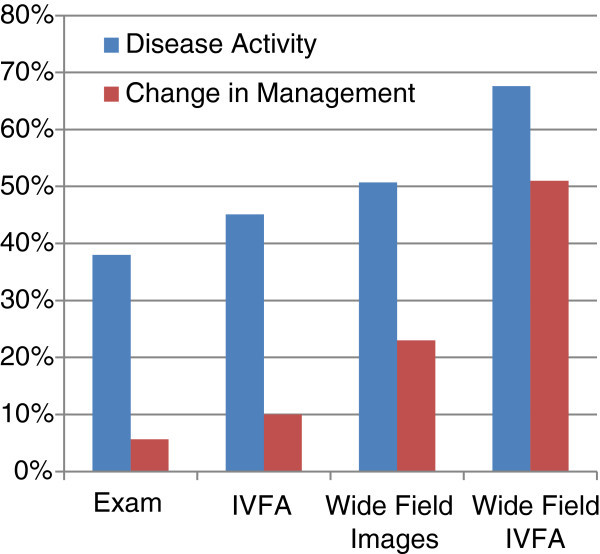
Active disease and change in management with increasing information.

Table 1 and Figure 2 depict the percentage of patients who demonstrated disease activity and whether management was changed based on the four possible combinations of examination and imaging. Disease activity was detected in 27/71 (38%) of patient visits based on clinical examination alone and an additional 5 of 71 (45%) based on simulated conventional FA. The addition of ultra-wide-field SLO pseudo-color images detected disease activity in 4 additional patient visits (51%), and ultra-wide-field FA identified an additional 12 patient visits (68%) as ‘active’ in whom neither clinical examination, standard FA, or even ultra-wide-field photographs had convincingly demonstrated disease activity. Based on the clinical examination alone, the decision to alter management was made in 4 of 71 visits (6%), and an additional 3 of 71 (4%) based on simulated standard FA. Thus, the investigator made the decision to alter management in 7 of 71 (10%) of patient visits based on examination and standard FA. The addition of ultra-wide-field SLO pseudo-color images altered management in 17/71 visits (24%), and the addition of ultra-wide-field FA altered management in 36/71 (51%) of patients.

**Table 1:** Percentage of patients who demonstrated disease activity and whether management was changed based on examination and imaging

	**Disease activity (*n*** **= 71) (%)**	***P***	**Change in management (*n*** **= 71) (%)**	***P***
Exam	38.02		5.63	
+FA	45.07	0.49^a^	10	0.47^a^
+Wide-field images	50.70	0.20^a^	24	0.01^a^
+Wide-field FA	67.60	0.0006^a^ and 0.0095^b^	51	<0.0001^a^ and <0.0001^b^

^a^Vs. clinical examination alone; ^b^vs. examination and FA.

## Discussion

In the index, prospective, observational study of patients with non-infectious vasculitis, the first, to our knowledge, to evaluate systematically and prospectively the role of ultra-wide-field imaging in retinal vasculitis, our results suggest that ultra-wide-field imaging may alter management decisions compared to standard-of-care imaging and clinical examination. Such differences are likely due to the ability to image the peripheral retinal and angiographic findings that are not easily visualized or identified without ultra-wide-field imaging. Both the determination of disease ‘activity’ and disease management differed significantly with the use of ultra-wide-field imaging compared with standard imaging. Management decisions were significantly altered, suggesting that qualitative and quantitative differences in the degree of disease activity seen on ultra-wide-field imaging may play an important role.

Our study was designed to investigate the potentials of wide-angle imaging in making management decisions in patients with non-infectious vasculitis, but not to determine the specific changes in patient management. Therefore, we did not obtain data on the management decisions that were made. Future investigations are needed to evaluate the degree of alteration (i.e., changing the dose of prednisone, addition of immunomodulatory therapy) associated with the additional information provided by the wide-angle imaging system and whether such alterations have led to additional benefits to the patients.

There are limitations to our study. The examining clinicians were not masked to 60°, as would have been ideal. Given the demands of clinical care and for practical and ethical reasons, we were unable to perform both the standard FA and the ultra-wide-field FA at the same visit. Thus, to determine disease activity and management based on examination and ‘conventional’ (30° or 60°) FA, investigators were asked to limit their assessment to the central 30° or 60° (based on preference) of the ultra-wide-field images (simulated conventional FA). Simulated peripheral sweeps were permitted if the investigator indicated that based on their clinical examination, peripheral sweeps were needed. The clinicians had to restrict themselves to the central image to evaluate the patient before considering the entire image. We attempted to limit bias by prospectively evaluating the ultra-wide-field imaging and acquiring the activity and management data based on the clinical examination prior to sending the patient for imaging, though the possibility of investigators biasing their responses to the standard FA question cannot be excluded with our study design.

In addition, it is also possible that there is selection bias in this study population towards the investigators selecting patients who may be more likely to have peripheral retinal findings that would change management, and not be representative of all patients with retinal vasculitis. Future studies may choose to enroll all patients with posterior uveitis consecutively to eliminate this potential bias.

In addition, ‘retinal vasculitis’ is a difficult entity to define. The Standardization of Uveitis Nomenclature Working Group [1] was unable to agree on a definition of vasculitis. We defined vasculitis as a retinal vascular occlusion, with leakage on fluorescein, overlying vitritis, vascular sheathing, and retinal hemorrhages in the context of ophthalmic inflammation. The definition of retinal vasculitis remains an area of debate, and different clinicians may define vasculitis using different criteria.

The index study was not designed to determine the utility of FA (standard or ultra-wide-field) in patients who do not appear to have active disease, though we did note a number of patients who demonstrated evidence of disease activity on peripheral angiography and who clinically appeared to have quiescent disease. The clinical significance of this ‘additionally noted’ disease activity is unknown since ultra-wide-field imaging modality has not previously been used in clinical practice or clinical trials.

## Conclusions

Our study represents the first attempt to investigate prospectively the use of ultra-wide-field imaging and angiography in patients with non-infectious retinal vasculitis. The findings from our study suggest that the additional information provided by ultra-wide-field imaging may allow earlier detection of active vasculitis, which may lead to earlier treatment and perhaps better patient outcomes. Ultra-wide-field imaging altered management decisions compared to standard-of-care imaging and/or clinical examination of patients with retinal vasculitis. The decision to alter management may be due to the ability of such systems to detect disease activity in the periphery, such as angiographic findings, not easily visualized or identified with examination or standard imaging alone. Additional longitudinal studies with larger patient populations are required to determine the utility and application of ultra-wide-field imaging in the management of patients with retinal vasculitis. It is possible that such studies will demonstrate improved patient outcomes with clinical management based on findings detected on ultra-wide-field photography and angiography.

## Methods

**Figure 3:**
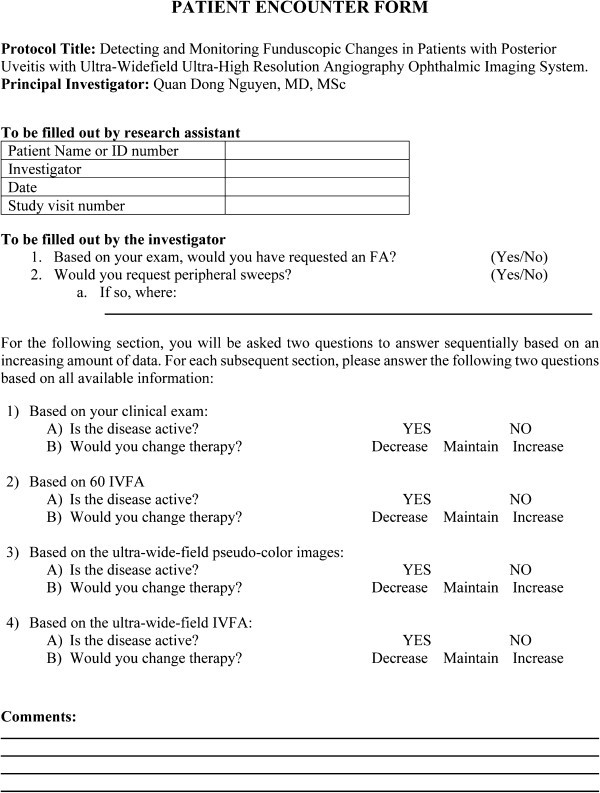
Data sheet for clinical decision making.

In this prospective, observational study of patients with non-infectious retinal vasculitis, disease activity and changes in management were assessed based on clinical examination with or without standard (60°) imaging and then with the addition of ultra-wide-field pseudo-color SLO images and FA using the Optos ultra-wide-field SLO (Optos Panoramic 200MA™, Optos PLC). New and established patients in the Divisions of Retina and Ocular Immunology at the Wilmer Eye Institute were evaluated, screened, and recruited by four co-investigators. A standardized questionnaire (Figure 3) was completed by each investigator at the time of the clinical evaluation. For practical and ethical reasons, we were unable to perform both the standard FA and the ultra-wide-field FA at the same visit. Thus, to determine disease activity and management based on examination and standard (60°) FA, investigators were asked to limit their assessment to the central 60° of the ultra-wide-field images. Simulated peripheral sweeps were permitted if the investigator indicated that based on their clinical examination, peripheral sweeps were needed. The images were evaluated for signs of activity, which include but are not limited to hemorrhages, vascular occlusions, perivascular sheathing, and leakage. The presence or absence of these signs was noted and compared to previous images when applicable.

The primary outcome was the percentage of patients whose management was changed by clinical exam and standard FA, compared with examination plus ultra-wide-field imaging. The secondary outcome was the percentage of patients whose disease was determined to be active with each modality. The primary and secondary outcome measures were analyzed in terms of proportions (i.e., proportion of patients who were determined to be active based on ultra-wide-field imaging compared with examination and standard FA) using the pretest function in Stata v11.0 (College Station, TX, USA).

The Institutional Review Board approval was obtained from the Johns Hopkins University School of Medicine before the study enrolled any patients. Written, informed consent was obtained from all patients included in this study. The research adhered to the tenets of the Declaration of Helsinki and was in accordance with all local, state, and federal regulations, including HIPAA.

## Competing interest

The research was made possible in part through the instrument support program from Optos, Inc. to the Johns Hopkins University. There was no direct financial support for this research from Optos.

## Authors’ contributions

HL, JC, and QN designed the study. HL, YS, TG, EH, BC, MI, RC, DD, JD, MB, and QN conducted the study. YS, HL, and QN managed, analyzed, and interpreted of the data. HL, DD, and QN prepared, reviewed, and approved the manuscript. All authors read and approved the final manuscript.

## References

[B1] Jabs DA Nussenblatt RB Rosenbaum JT Standardization of Uveitis Nomenclature (SUN) Working Group Standardization of uveitis nomenclature for reporting clinical data. Results of the First International Workshop Am J Ophthalmol 2005 140 509 516 16196117 10.1016/j.ajo.2005.03.057 PMC8935739

[B2] Manivannan A Plskova J Farrow A Mckay S Sharp PF Forrester JV Ultra-wide field fluorescein angiography of the ocular fundus Am J Ophthalmol 2005 140 525 527 10.1016/j.ajo.2005.02.055 16139004

[B3] Kaines A Tsui I Sarraf D Schwartz S The use of ultra wide field fluorescein angiography in evaluation and management of uveitis Semin Ophthalmol 2009 24 19 24 10.1080/08820530802520095 19241287

[B4] Anderson L Friberg TR Singh J Ultrawide-angle retinal imaging and retinal detachment Semin Ophthalmol 2007 22 43 47 10.1080/08820530601162867 17366119

[B5] Coffee RE Jain A McCannel TA Ultra wide field imaging of choroidal metastasis secondary to primary breast cancer Semin Ophthalmol 2009 24 34 36 10.1080/08820530802520194 19241290

[B6] Jain A Shah SP Tsui I McCannel TA The value of Optos Panoramic 200MA imaging for the monitoring of large suspicious choroidal lesions Semin Ophthalmol 2009 24 43 44 10.1080/08820530802520384 19241293

[B7] Kernt M Pinter F Hadi I Hirneiss C Haritoglou C Kampik A Ulbig MW Neubauer AS Diabetic retinopathy: comparison of the diagnostic features of ultra-widefield scanning laser ophthalmoscopy Optomap with ETDRS 7-field fundus photography Ophthalmologe 2011 108 117 123 10.1007/s00347-010-2226-4 20683601

[B8] Kernt M Schaller UC Stumpf C Ulbig MW Kampik A Neubauer AS Choroidal pigmented lesions imaged by ultra-wide field scanning laser ophthalmoscopy with two laser wavelengths (Optomap) Clin Ophthalmol 2010 4 829 836 20689737 10.2147/opth.s11864 PMC2915871

[B9] Khandhadia S Madhusudhana KC Kostakou A Forrester JV Newsom RS Use of Optomap for retinal screening within an eye casualty setting Br J Ophthalmol 2009 93 52 55 10.1136/bjo.2008.148072 18971233

[B10] Mackenzie PJ Russell M Ma PE Isbister CM Maberley DA Sensitivity and specificity of the Optos Optomap for detecting peripheral retinal lesions Retina 2007 27 1119 1124 10.1097/IAE.0b013e3180592b5c 18040256

[B11] Meyer CH Saxena S Non-mydriatic imaging of a giant retinal tear with the Optos Optomap Panoramic 200MA Clin Experiment Ophthalmol 2010 38 427 10.1111/j.1442-9071.2010.02260.x 20642589

[B12] Mudvari SS Virasch VV Singa RM MacCumber MW Ultra-wide field imaging for cytomegalovirus retinitis Ophthalmic Surg Lasers Imaging 2010 41 311 315 10.3928/15428877-20100430-03 20507014

[B13] Prasad PS Oliver SC Coffee RE Hubschman JP Schwartz SD Ultra wide field angiographic characteristics of branch retinal and hemicentral retinal vein occlusion Ophthalmology 2010 117 780 784 10.1016/j.ophtha.2009.09.019 20045570

[B14] Shah SP Jain A Tsui I McCannel TA Optos Optomap Panoramic 200MA imaging of a serous choroidal detachment responsive to furosemide Semin Ophthalmol 2009 24 40 42 10.1080/08820530802520236 19241292

[B15] Spaide RF Peripheral areas of nonperfusion in treated central retinal vein occlusion as imaged by wide-field fluorescein angiography Retina 2011 31 5 829 837 10.1097/IAE.0b013e31820c841e 21487338

